# Trends over the recent 6 years in ablation modalities and strategies, post‐ablation medication, and clinical outcomes of atrial fibrillation ablation

**DOI:** 10.1002/joa3.12854

**Published:** 2023-04-23

**Authors:** Moyuru Hirata, Koichi Nagashima, Ryuta Watanabe, Yuji Wakamatsu, Naoto Otsuka, Satoshi Hayashida, Shu Hirata, Masanaru Sawada, Sayaka Kurokawa, Yasuo Okumura

**Affiliations:** ^1^ Division of Cardiology, Department of Medicine Nihon University School of Medicine Tokyo Japan

**Keywords:** atrial fibrillation, catheter ablation, clinical outcome

## Abstract

**Background:**

Ablation strategies and modalities for atrial fibrillation (AF) have transitioned over the past decade, but their impact on post‐ablation medication and clinical outcomes remains to be fully investigated.

**Methods:**

We divided 682 patients who had undergone AF ablation in 2014–2019 (420 paroxysmal AFs [PAF], 262 persistent AFs [PerAF]) into three groups according to the period, that is, the 2014–2015 (*n* = 139), 2016–2017 (*n* = 244), and 2018–2019 groups (*n* = 299), respectively.

**Results:**

Persistent AF became more prevalent and the left atrial (LA) diameter larger over the 6 years. Extra‐pulmonary vein (PV)‐LA ablation was more frequently performed in the 2014–2015 group than in the 2016–2017 and 2018–2019 groups (41.1% vs. 9.1% and 8.1%; *p* < .001). The 2‐year freedom rate from AF/atrial tachycardias for PAF was similar among the three groups (84.0% vs. 83.1% vs. 86.7%; *p* = .98) but lowest in the 2014–2015 group for PerAF (63.9% vs. 82.7% and 86.3%; *p* = .025) despite the highest post‐ablation antiarrhythmic drug use. Cardiac tamponade was significantly decreased in the 2018–2019 group (3.6% vs. 2.0% vs. 0.33%; *p* = 0.021). There was no difference in the 2‐year clinically relevant events among the three groups.

**Conclusion:**

Although ablation was performed in a more diseased LA and extra‐PV‐LA ablation was less frequent in recent years, the complication rate decreased, and AF recurrences for PAF remained unchanged, but that for PerAF decreased. Clinically relevant events remained unchanged over the recent 6 years, suggesting that the impact of the recent ablation modalities and strategies on remote clinically relevant events may be small during this study period.

## Graphical abstract

Although ablation was performed in a more diseased left atrium (LA) and extra‐PV LA ablation was less frequent in recent years, the complication rate decreased, and AF recurrences for PAF remained unchanged, but that for PerAF decreased.

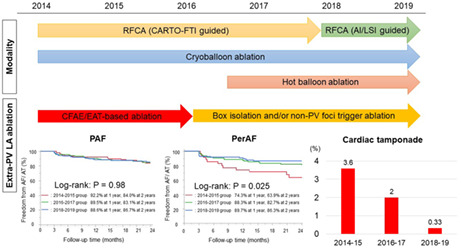

## 1 INTRODUCTION

Recently, catheter ablation of atrial fibrillation (AF) has become a widely accepted therapy because it has rigid evidence of maintaining sinus rhythm over antiarrhythmic therapy.^1^, ^2^ Pulmonary vein isolation (PVI) has been the standard ablation strategy for paroxysmal AF (PAF), and atrial substrate modification such as complex fractionated atrial electrogram (CFAE) ablation, linear ablation, and/or ablation of non‐PV triggers had been one of the relevant clinical interests for improving AF ablation in patients with a remodeled atrium including persistent AF (PerAF) and long‐lasting PerAF over the past decade.^3^, ^4^, ^5^ Nonetheless, the Substrate and Trigger Ablation for Reduction of Atrial Fibrillation Trial Part II (STAR‐AF2) demonstrated that substrate modification did not reduce the recurrence rate of AF.^5^ Three‐dimensional mapping and ablation technology for AF ablation has been developed, that is, contact force and the force–time integral (FTI),^6^, ^7^ and has been followed by the ablation index (AI) and lesion size index (LSI), which have been incorporated into both the CARTO3 and Ensite NavX mapping systems to create an appropriate lesion formation.^8^, ^9^, ^10^, ^11^, ^12^, ^13^ The balloon‐based technologies such as cryoballoon ablation (CBA) and hot balloon ablation (HBA) also have been launched, and the efficacy and safety of those balloon‐based ablation systems have been reported in previous studies.^14^, ^15^, ^16^, ^17^ Recently, the CABANA and CASTLE‐AF trials demonstrated that ablation reduces long‐term rehospitalizations because of heart failure (HF) and all‐cause mortality mainly driven by the maintenance of sinus rhythm by ablation,^18^, ^19^ which may promote more physicians to perform AF ablation throughout the world. This trend may also motivate physicians to confidently terminate antiarrhythmic drugs (AADs) and oral anticoagulants (OACs) after ablation. Despite these transitions in the strategies and modalities, their effects on the post‐ablation AADs and OACs and subsequent long‐term outcomes in real‐world practice, remain not fully investigated. Therefore, this study aimed to examine the characteristics of the patients who underwent AF ablation, the transition of the strategies and modalities for AF ablation, and the 2‐year success rate and clinical adverse outcomes after AF ablation in the recent 6 years from 2014 to 2019.

## 2 METHODS

### 2.1 Study design

This retrospective observational study included 682 consecutive patients who underwent an initial AF ablation at Nihon University Itabashi Hospital between 2014 and 2019. We divided the 682 patients into three time period groups, that is, patients who underwent ablation from January 2014 to December 2015 (2014–2015 group), from January 2016 to December 2017 (2016–2017 group), and from January 2018 to December 2019 (2018–2019 group), respectively.

### 2.2 Data collection

The data collection has been described elsewhere.^20^, ^21^ In brief, the patient characteristics and pre‐ablation (baseline) and follow‐up data were obtained through a review of their hospital charts. The pseudonymized patient data were collected in an Excel format by physicians, and it included the patient characteristics such as the gender, age, body mass index (BMI), type of AF, any comorbidity (hypertension, diabetes, HF, history of a stroke/transient ischemic attack [TIA]), transthoracic echocardiography‐derived left atrial diameter (LAd) and left ventricular ejection fraction (LVEF), type of pre ablation AADs, strategies of AF ablation (extra‐PV left atrial [LA] ablation and/or cavotricuspid isthmus [CTI] ablation), and the modalities of AF ablation (radiofrequency [RF], balloon ablation, or use of the FTI, AI, or LSI). The follow‐up variables included the post‐ablation usage of AADs and any OACs, 2‐year freedom rate from AF or atrial tachycardia (AT) after a blanking period of 3 months, and occurrence of any clinically relevant events such as hospitalizations because of HF, major bleeding, and cardiovascular events, and all‐cause mortality after catheter ablation.

### 2.3 AF ablation protocol

**FIGURE 1:**
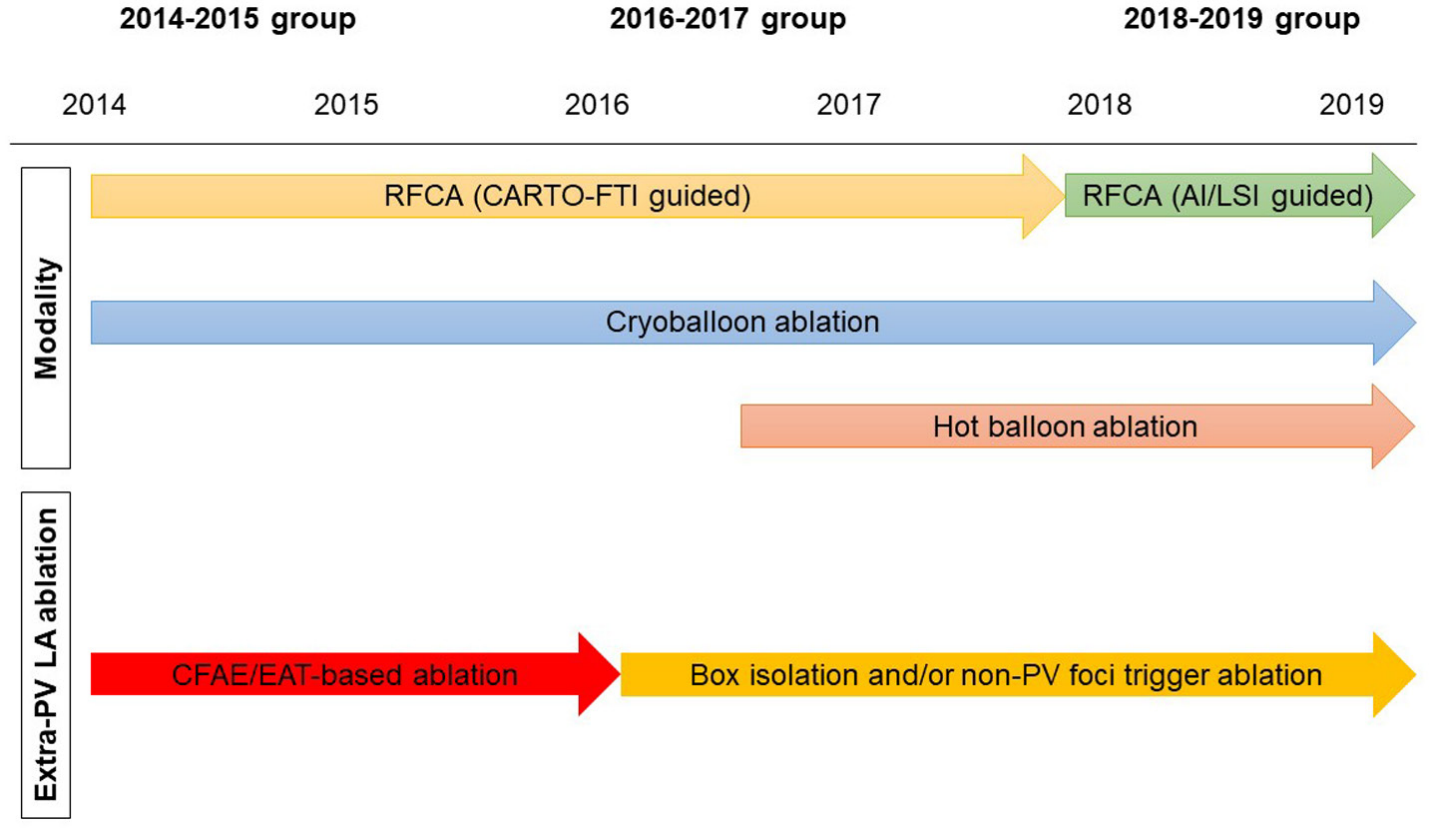
Ablation modality and strategy over 6 years. AI, ablation index; CFAE, complex fractionated atrial electrogram; EAT, epicardial tissue; FTI, force–time integral; LSI, lesion index.

Before the electrophysiologic study, all AADs were discontinued for at least five half‐lives. The analysis was performed with patients under conscious sedation with dexmedetomidine and fentanyl. After a single transseptal puncture was performed, two long sheaths (Agilis steerable sheath and SL0 sheath [Abbott, Inc.]) were inserted into the LA via a transseptal puncture. An activated clotting time > 300 s was maintained by heparin during the procedure. The 3D geometry of the LA and PVs was created using a CARTO3 (Biosense Webster, Diamond Bar, CA) or Ensite NavX mapping system (Abbott). The transition of the modalities and strategy of the catheter ablation of AF are shown in Figure 1. As for RF ablation, an extensive encircling PVI was performed with an irrigated‐tip contact force RF ablation catheter (3.5‐mm tip, Navistar ThermoCool SmartTouch, Biosense Webster; or TactiCath, Abbott) under the guidance of the FTI in 2014–2017, and then the AI or LSI in 2018–2019. The target FTI was 450 for the anterior sites and 350–400 for the posterior sites with a power setting of 30–35 W,^7^ and the target AI and LSI were 450 and 5.5 for the anterior sites and 400 and 4.5–5.0 for the posterior sites with a power of 40‐45 W, respectively.^12^ Extra‐PV‐LA ablation including a CFAE/epicardial adipose tissue (EAT)‐guided ablation in the LA,^22^, ^23^ and linear ablation and/or non‐PV foci ablation was performed in cases in which AF was sustained even after the PVI depending on the operator's discretion. The CFAE/EAT‐guided ablation in the LA was performed especially during the past 2014–2015 year, and linear ablation and/or non‐PV foci ablation was mainly performed thereafter (Figure 1).

Regarding the balloon‐based ablation, the Ensite NavX mapping system was used to guide the LA‐PV mapping. Since 2014, CBA was performed, as reported previously.^15^, ^16^, ^17^ A 28 mm cryoballoon (ARC‐Adv‐CB, Arctic Front Advance; Medtronic, Inc) was used and geothermal energy was applied to each PV for 180 s and then for 120 s. Since 2017, HBA started to be performed as reported previously.^15^, ^16^ In brief, a hot balloon (SATAKE HotBalloon; Toray Industries, Inc) using radiofrequency‐generated thermal energy was applied to the right superior PV antrum for 210 s, left superior PV antrum for 240 s, and right and left inferior PV antra for 150 s. In both CBA and HBA, no extra‐PV‐LA ablation was performed. Regardless of the use of RF ablation, CBA, or HBA, a CTI ablation was performed in cases in which atrial flutter was clinically documented or incidentally lasted for 1 min during the procedure.

### 2.4 Follow‐up

AADs were resumed after the ablation procedure at the operator's decision. All patients underwent routine follow‐up at our institution at 3 weeks and 3, 6, 12, and 24 months after ablation or whenever they had any symptoms. Twelve‐lead electrograms were recorded at each visit, and 24‐h Holter recordings were obtained at 3, 6, 12, and 24 months after the ablation procedure. The patients were generally introduced to other private clinics after 3 months post‐ablation and were followed up every 1–3 months thereafter. Recurrence was defined as any document of AF or AT of more than 30 s during 3–24 months after the ablation.

### 2.5 Study end points

The primary efficacy end point was the freedom from AF or AT recurrence, and the primary safety end point was ablation‐related complications during the post‐procedural period (within 1 month after ablation). Secondary end points were clinically relevant events including a stroke/TIA, hospitalization because of HF, major bleeding, and cardiovascular events, and all‐cause mortality.

### 2.6 Statistical analysis

Continuous variables are expressed as the mean ± SD values or median and interquartile range, and categorical variables are defined as the number and percentage of patients. A Student's *t*‐test or Mann–Whitney U test was used, as appropriate, to analyze the differences in the continuous variables, and a chi‐square test was used to analyze the differences in the dichotomous variables unless the expected values in the cells were <5 in which case a Fisher's exact test was used. Basically, the *p*‐value among the three groups was calculated by an ANOVA or chi‐square test followed by the Tukey‐HSD test or post hoc Bonferroni test. The 2‐year continuation rate of AADs and OACs, freedom from an AF/ AT recurrence, and clinically relevant events were compared among the groups by a Kaplan–Meier analysis with a log‐rank test in the PAF and PerAF patients, respectively. Also, the 2‐year cumulative rate of clinically relevant events was compared between the high‐risk and low‐risk patients by a Kaplan–Meier analysis with a log‐rank test. Patients were censored at the time of the first discontinuation of the AADs and OACs. A univariate cox regression analysis was performed to identify the clinical indicators associated with post‐ablation AT/AF recurrences. The three period groups, age, gender, CHA_2_DS_2_‐VASc score, and extra‐PV‐LA ablation were entered into the multivariate model for Per AF. All previous statistical analyses were performed with JMP Pro 16 software (SAS Institute). A *p* <.05 was considered statistically significant.

## 3 RESULTS

### 3.1 Patient characteristics, modality, and strategy among the three time period groups based on the date of the AF ablation

The characteristics, and ablation modality and strategy among the 2014–2015, 2016–2017, and 2018–2019 groups are shown in Tables 1 and 2. The age gradually became older (62.6 ± 10.5 vs. 63.6 ± 10.2 vs. 65.7 ± 10.3 years; *p* = .006 by ANOVA) and persistent AF more prevalent (25.2% vs. 43.0% vs. 40.8%; *p* = .001), LVEF lower (67.6 ± 8.4% vs. 65.5 ± 10.8% vs. 64.5 ± 9.8%; *p* = .01), LAd larger (38.5 ± 5.8 mm vs. 39.5 ± 6.5 mm vs. 40.9 ± 6.7 mm; *p* = .001), and DM and HF tended to be more prevalent from the 2014–2015 group, to the 2016–2017 group, and to the 2018–2019 group, respectively. There were no significant differences in the number of high‐risk patients with a CHADS_2_ score ≥2 (33.8% vs. 34.0% vs. 36.4%; *p* = .79) and CHA_2_DS_2_‐VASc score ≥3 (33.8% vs. 34.8% vs. 40.8%; *p* = .23) between the three time period groups. Among the pre‐ablation AADs, the class I AADs decreased, and the β blockers gradually increased from the 2014–2015 group, to the 2016–2017 group, and to the 2018–2019 group, respectively (*p* < .001 for both). Balloon ablation was most frequently performed in the 2016–2017 group (30% vs. 64.3% vs. 48.2%; *p* < .001). Extra‐PV‐LA ablation was most frequently performed in the 2014–2015 group as compared to the 2016–2017 group and 2018–2019 group (41.1% vs. 9.1% vs. 8.1%; *p* < .001), and likewise, a CTI ablation was also performed (43.1% vs. 34.4% vs. 23.4%; *p* < .001). The patient characteristics among the three time period groups in the PAF and PerAF patients are shown in Table S1. The overall trend in the patient characteristics was similar regardless of PAF or PerAF.

**TABLE 1:** Patient characteristics, ablation modalities, and strategies between the three time‐year period groups.

	2014–2015 group (*n* = 139)	2016–2017 group (*n* = 244)	2018–2019 group (*n* = 299)	*p*‐value
Age (years)	62.6 ± 10.5	63.6 ± 10.2	65.7 ± 10.3*	.006
Male gender	93 (66.9%)	179 (73.3%)	212 (70.9%)	.40
BMI (kg/m^2^)	23.9 ± 4.0	24.6 ± 4.0	24.2 ± 3.7	.27
Persistent AF	35 (25.2%)	105 (43.0%)*	122 (40.8%)*	.001
Medical history				
HT	80 (57.6%)	146 (59.8%)	168 (56.2%)	.69
DM	19 (13.6%)	50 (20.5%)	40 (13.4%)	.06
HF	10 (7.2%)	33 (13.5%)	44 (14.7%)	.08
Vascular disease	8 (5.7%)	16 (6.6%)	14 (4.7%)	.63
Stroke/TIA	15 (10.8%)	18 (7.4%)	27 (12.5%)	.52
CHADS_2_ score	1 (0, 2)	1 (0, 2)	1 (0, 2)	.41
CHADS_2_ score ≥2	47 (33.8%)	83 (34.0%)	109 (36.4%)	.79
CHA_2_DS_2_‐VASc score	2 (1, 3)	2 (1, 3)	2 (1, 3)	.58
CHA_2_DS_2_‐VASc score ≥3	47 (33.8%)	85 (34.8%)	122 (40.8%)	.23
Echocardiographic variables				
LVEF (%)	67.6 ± 8.4	65.5 ± 10.8	64.5 ± 9.8*	.010
LAd (mm)	38.5 ± 5.8	39.5 ± 6.5	40.9 ± 6.7* ^,^ **	.001
Pre‐ablation AADs	88 (63.3%)	99 (40.5%)*	137 (45.8%)*	<.001
Class I	49 (35.3%)	48 (19.7%)*	61 (20.4%)* ^,^ **	<.001
Class III	7 (5.0%)	10 (4.1%)	9 (3.0%)	.56
Bepridil	32 (23.0%)	41 (16.8%)	73 (24.4%)	.09
β blocker	27 (19.4%)	77 (31.6%)*	129 (43.1%)* ^,^ **	<.001

*Note*: The mean ± SD values or number (%) of patients are shown.

Abbreviations: AADs, antiarrhythmic drugs; AF, atrial fibrillation; BMI, body mass index; DM, diabetes mellitus; HF, heart failure; HT, hypertension; LAD, left atrial diameter; LVEF, left ventricular ejection fraction.

* *p* < 0.05 versus 2014–2015 group by the Tukey‐HSD test or post hoc Bonferroni test

** *p* < 0.05 versus 2016–2017 group by the Tukey‐HSD test or post hoc Bonferroni test.

**TABLE 2:** AF ablation modalities and strategies between the three time‐year period groups among the total study patients, paroxysmal AF patients, and persistent AF patients.

All patients	2014–2015 group	2016–2017 group	2018–2019 group	
Total no.: *n* = 682	*n* = 139	*n* = 244	*n* = 299	*p* value
Modality				
RFCA	97 (70.0%)	87 (35.7%)*	155 (51.8%)*	<.001
Balloon ablation	42 (30.0%)	157 (64.3%)*	144 (48.2%)*	<.001
Strategy				
Extra‐PV‐LA ablation	57 (41.1%)	22 (9.1%)*	54 (8.1%)*	<.001
CTI ablation	60 (43.1%)	84 (34.4%)*	70 (23.4%)*	<.001

Paroxysmal AF				
Total no.: *n* = 420	*n* = 104	*n* = 139	*n* = 177	*p* value
Modality				
RFCA	70 (67.3%)	49 (35.2%)*	78 (44.1%)*	<.001
Balloon ablation	34 (32.7%)	90 (64.8%)*	99 (55.9%)*	<.001
Strategy				
Extra‐PV‐LA ablation	36 (34.6%)	13 (9.3%)*	14 (7.9%)*	<.001
CTI ablation	42 (40.3%)	46 (33.9%)	46 (25.9%)*	.041

Persistent AF				
Total no.: *n* = 262	*n* = 35	*n* = 105	*n* = 122	*p* value
Modality				
RFCA	27 (77.1%)	38 (36.1%)*	77 (63.1%)**	
Balloon ablation	8 (22.9%)	67 (63.8%)*	45 (36.9%)**	<.001
Strategy				
Extra‐PV‐LA ablation	21 (60.0%)	9 (8.5%)*	40 (32.8%)*, ^,^**	<.001
CTI ablation	18 (51.4%)	38 (36.1%)	24 (19.6%)**	<.001

*Note*: The number (%) is shown.

Abbreviations: AF, atrial fibrillation; CTI, cavotricuspid isthmus; LA, left atrium; PV, pulmonary vein; RFCA, radiofrequency catheter ablation.

* *p* < 0.05 versus 2014–2015 group by the Tukey‐HSD test or post hoc Bonferroni test

** *p* < 0.05 versus 2016–2017 group by the Tukey‐HSD test or post hoc Bonferroni test.

### 3.2 Complications and clinical outcomes

**FIGURE 2:**
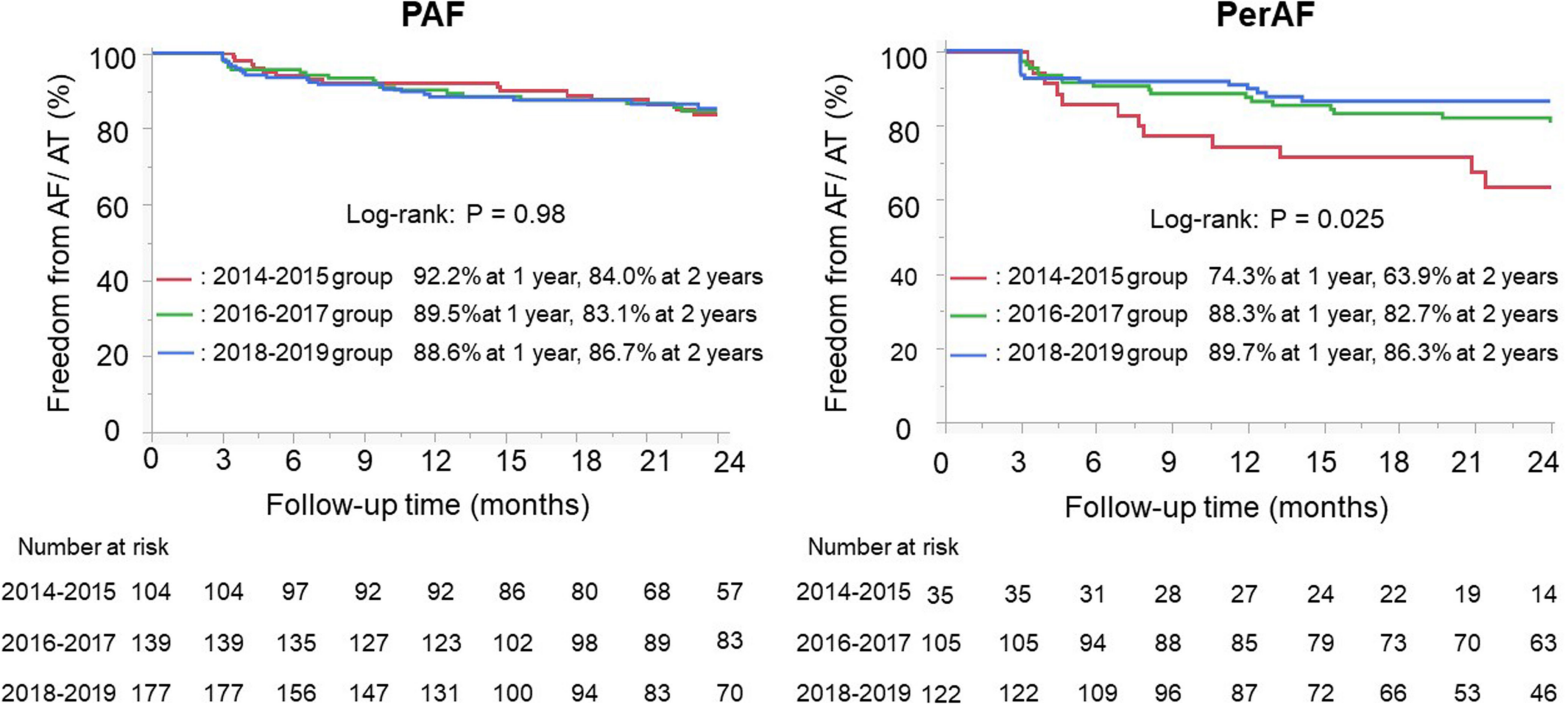
Kaplan–Meier curves for the freedom from AF/AT after an initial ablation among the 2014–2015, 2016–2017, and 2018–2019 groups.

The complications associated with AF ablation are shown in Table 3. Cardiac tamponade decreased significantly more in the 2018–2019 group than in the other time period groups (0.33% vs. 3.6% in the 2014–2015 group and 2.0% in the 2016–2017, respectively; *p* = .021). There was no significant difference in other complications of AF ablation among the three groups. The 2‐year continuation rate of AADs for PAF based on the Kaplan–Meier curve was significantly lower in the 2016–2017 group than in the other time periods (17.0% vs. 25.4% in the 2014–2015 group and 27.0% in the 2018–2019 group; *p* = .013 by log‐rank test), but that rate in the PerAF patients was significantly lower in the 2016–2017 and 2018–2019 groups than in the 2014–2015 group (37.0% and 44.8% vs. 63.8%; *p* = .002) (Figure S1). Of a total of 179 patients who had discontinued the post‐ablation AADs during the 2‐year follow‐up, only two (1.1%) had undergone a repeat session. The 2‐year OACs continuation rate in the PAF and PerAF patients based on the Kaplan–Meier curve was the highest in the 2016–2017 group (PAF 60.2% vs. 41.5% in the 2014–2015 group and 50.7% in the 2018–2019 group; *p* = .09: PerAF 73.5% vs. 43.2% in the 2014–2015 group and 47.4% in the 2018–2019 group; *p* < .001) (Figure S1). Of a total of 313 patients who had discontinued OACs during the 2‐year follow‐up, only two (0.6%) had undergone a repeat session. The 2‐year freedom rate from AF/AT in the PAF and PerAF patients is shown in Figure 2. The 2‐year AF/AT freedom rate of PAF did not differ among the three time period groups (84.0% vs. 83.1% vs. 86.7%; *p* = .98 by log‐rank test), however, the 2‐year AF/AT freedom rate of PerAF was significantly lower in the 2014–2015 group than in the 2016–2017 and 2018–2019 groups (63.9% vs. 82.7% and 86.3%; *p* = .025). The Cox regression analysis for determining the clinical variables associated with post‐ablation AF/AT recurrences in the PAF group and PerAF group is described in Table 4. In the PAF group, the univariate analysis did not identify any significant clinical variables associated with AF/AT recurrences. In the PerAF group, the univariate analysis identified a significant risk reduction in the 2016–2017 and 2018–2019 groups relative to the 2014–2015 group as a counterpart. After a multivariate adjustment, only the 2018–2019 group was independently associated with AF/AT recurrences (HR 0.38 vs. 2014–2015 group, 95% CI 0.19–0.96; *p* = .020) (Table 4).

**TABLE 3:** Complications of catheter ablation of AF.

	2014–2015 group (*n* = 139)	2016–2017 group (*n* = 244)	2018–2019 group (*n* = 299)	*p* value
Cardiac tamponade	5 (3.6%)	5 (2.0%)	1 (0.33%)*	.021
Stroke	0	1 (0.41%)	1 (0.33%)	.49
Sinus node dysfunction	2 (1.44%)	0	2 (0.67%)	.43
Phrenic nerve palsy	0	0	2 (0.29%)	.33
Gastrointestinal complication	2 (1.44%)	1 (0.41%)	2 (0.67%)	.19
Arteriovenous fistula	1 (0.72%)	2 (0.82%)	1 (0.33%)	.57
Pseudoaneurysm	2 (1.44%)	2 (0.82%)	1 (0.33%)	.19

*Note*: The number (%) of patients is shown.

Abbreviation: AF, atrial fibrillation.

* *p* < 0.05 versus 2014–2015 group by the post hoc Bonferroni test.

**TABLE 4:** Clinical variables associated with AF/AT recurrence after ablation in PAF group and PerAF group.

	PAF		PerAF			
	Univariate analysis		Univariate analysis		Multivariate analysis	
Variables	HR (95% CI)	*p*‐value	HR (95% CI)	*p*‐value	HR (95% CI)	*p*‐value
2014–2015 group	Reference	—	Reference	—	Reference	—
2016–2017 group	1.04 (0.53–2.04)	.89	0.48 (0.22–0.98)	.045	0.50 (0.20–1.12)	.11
2018–2019 group	0.99 (0.51–1.90)	.96	0.37 (0.17–0.79)	.011	0.38 (0.19–0.96)	.020
Age	0.99 (0.96–1.01)	.31	1,00 (0.97–1.02)	.82	1.01 (0.97–1.04)	.98
Male	0.84 (0.49–1.43)	.52	1.01 (0.49–2.03)	.98	0.83 (0.37–1.84)	.64
BMI	1.01 (0.94–1.07)	.68	1.00 (0.92–1.07)	.91	—	—
CHADS_2_ score	0.97 (0.74–1.24)	.81	0.85 (0.62–1.12)	.27	—	—
CHA_2_DS_2_‐VASc score	0.96 (0.79–1.14)	.65	0.89 (0.72–1.09)	.28	0.84 (0.64–1.10)	.21
HF	1.60 (0.76–3.38)	.21	0.76 (0.32–1.79)	.75	—	—
HT	1.148 (0.67–1.95)	.60	0.75 (0.41–1.35)	.33	—	—
DM	1.10 (0.55–2.17)	.78	1.00 (0.44–2.22)	.99	—	—
Stroke/TIA	0.61 (0.19–1.96)	.41	0.70 (0.21–2.25)	.52	—	—
EF	0.99 (0.96–1.01)	.29	1.00 (0.98–1.03)	.51	—	—
LAd	1.00 (0.96–1.04)	.89	1.00 (0.96–1.05)	.75	—	—
Post‐ablation AADs*	1.58 (0.90–2.73)	.10	2.79 (1.48–5.24)	.001	—	—
Post‐ablation OAC*	2.78 (1.51–5.07)	<.001	1.91 (0.98–3.70)	.044	—	—
Balloon ablation	0.89 (0.52–1.48)	.64	0.80 (0.44–1.45)	.46	—	—
AI/LSI‐guided ablation	0.94 (0.48–1.82)	.86	0.83 (0.41–1.63)	.58	—	—
Extra‐PV‐LA ablation	0.75 (0.35–1.58)	.43	1.42 (0.77–2.62)	.31	1.03 (0.48–2.16)	.94

Abbreviations: AT, atrial tachycardia; HR, hazard ratio. Other abbreviations as in Table 1.

* Medication use at the final follow‐up or at 2‐year after ablation.

**FIGURE 3:**
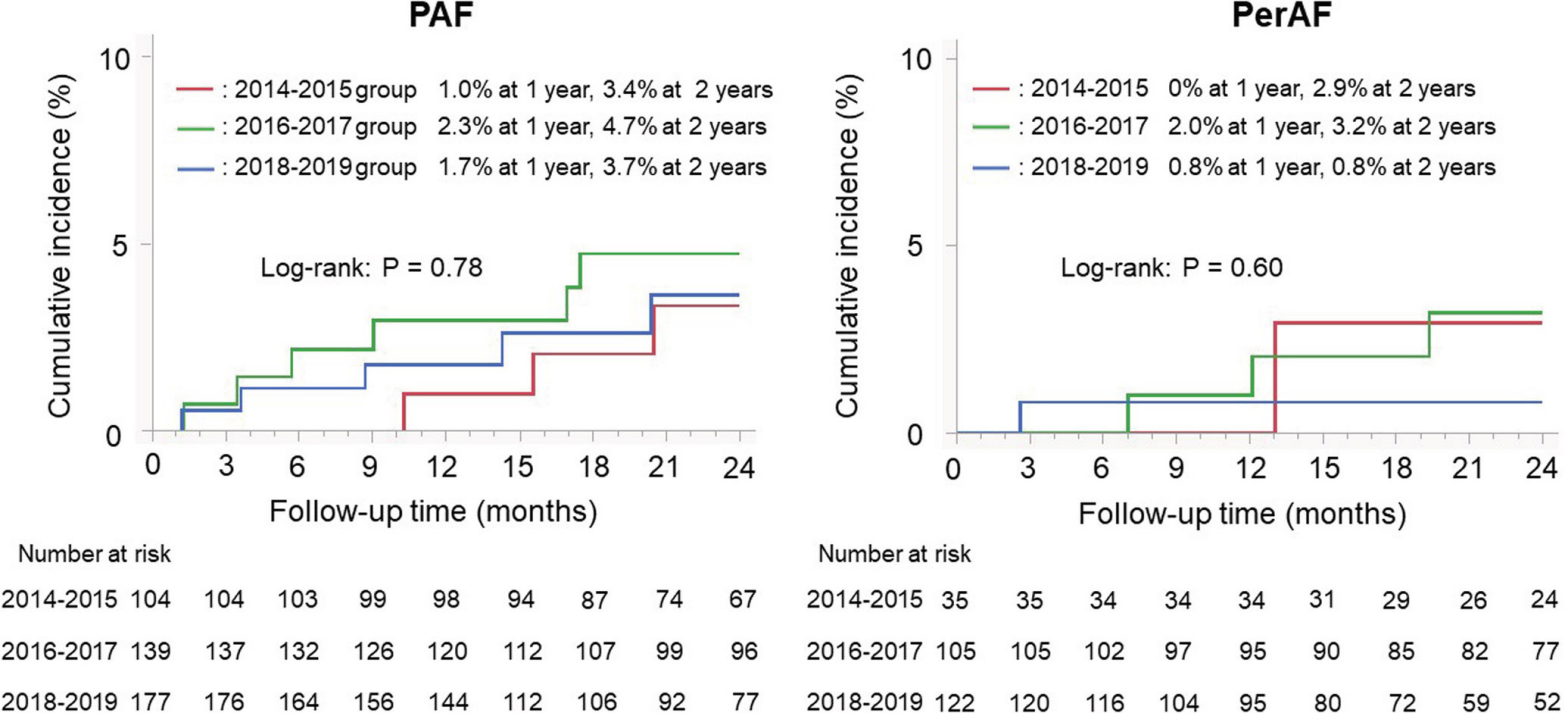
Kaplan–Meier curves for the cumulative incidence of clinically relevant events after an initial ablation among the 2014–2015, 2016–2017, and 2018–2019 groups.[Correction added on 29 April 2023 after first online publication: Figure 3 in the article is corrected in this version.]

The Kaplan–Meier curves for clinically relevant events in the PAF and PerAF patients are shown in Figure 3. There were no differences in the clinically relevant events among the three time period groups. The details of each adverse event are summarized in Table S2. No difference in each clinically relevant event was also found among the three time period groups. However, the 2‐year cumulative rate of clinically relevant events was significantly associated with high‐risk patients with a CHADS_2_ score ≥2 (6.2% vs. 1.8% in the low‐risk patients with 0 and 1; *p* = .008 by log‐rank test) and CHA_2_DS_2_‐VASc score ≥3 (5.8% vs. 1.8% in the low‐risk patients with <3; *p* = .018), respectively.

## 4 DISCUSSION

### 4.1 Main findings

This study had three major findings: (1) the patients were older, more frequently had persistent AF, developed a lower EF, and had a larger LAd for the more recent time periods. Balloon ablation was frequently performed in the latter two periods, (2) although the patients had a lesser remodeled LA substrate and extra‐PV‐LA ablation was frequently performed in the 2014–2015 group, the 2‐year‐recurrence rate was similar among the three periods for PAF but that rate was higher in the 2014–2015 group for PerAF, and (3) there were no differences in 2‐year clinically relevant events regardless of PAF or PerAF.

### 4.2 The time‐course changes in the patient characteristics, complications, and recurrence rate of AF/AT after ablation over the recent 6 years

These single‐center results showed that patients who had undergone AF ablation gradually became older, and had a larger LAd, lower EF, and more often PerAF and HF over the past 6 years. A higher efficacy in terms of sinus rhythm maintenance by ablation over AADs in symptomatic PAF has been widely established.^1^, ^2^, ^24^ Catheter ablation appears to be superior to medical therapy even in PerAF,^8^ in particular, in those with HF.^25^ Several randomized control trials have shown the benefit of ablation over medical therapy in terms of the quality of life and prognosis in patients with AF and HF associated with a reduced ejection fraction (HFrEF).^18^, ^26^ Accordingly, the guidelines over the world have recommended catheter ablation for symptomatic PAF patients as class I and symptomatic persistent AF and AF with HFrEF as class IIa or IIb.^27^, ^28^, ^29^ Our results reflected that increased evidence of AF ablation made physicians expand the patients for the indication of AF ablation. Recently, early intervention by ablation has been shown to be more beneficial for the maintenance of sinus rhythm and the prevention of AF progression than standard medical therapy.^30^, ^31^, ^32^, ^33^ The less frequent use of pre‐ablation AADs in the recent 4 years in PAF patients has suggested an early intervention by ablation in recent years. Regarding the ablation‐related complications, cardiac tamponade was significantly lower in the 2018–2019 group than in the other time period groups. Ablation formation optimized by the AI or LSI might significantly confer favorable effects on those complications.^34^

In PAF patients, the 2‐year AF/AT freedom rate was similarly high among the three time period groups. PAF patients became older and the LAd larger over those 6 years, suggesting PAF patients with more remodeled substrates were included in the recent years. The post‐ablation use of AADs might be a confounder; however, those were not statistically associated with AF/AT recurrence. Several meta‐analysis reports have shown a contact force‐guided PVI and balloon‐based ablation significantly improved the AF freedom rate.^35^, ^36^ The PAF patients in this study underwent a contact force‐guided PVI or balloon ablation. Therefore, our results might support a high success rate with PVI methods achieving a durable PVI in PAF patients even when extending the ablation indication to a remodeled LA.

In contrast, we found an important finding that the 2‐year AF/AT freedom rate in PerAF patients was less frequent, however, PerAF patients also tended to have more remodeled substrates in the two recent time period groups, as seen with PAF patients. Regarding the ablation modality and strategy, extra‐PV‐LA ablation with a contact force‐guided PVI followed by post‐ablation AADs was the highest in the 2014–2015 group, while in the 2018–2019 group, a box isolation with a contact force‐guided PVI was performed in one third of the patients, and post‐ablation AADs were less frequently used as seen in the 2014–2015 group. Important to note, in the 2016–2017 group, balloon ablation was the most frequent, and extra‐PV‐LA ablation and the use of post‐ablation AADs were the lowest. A multivariate analysis revealed that the 2018–2019 group had a significantly lower incidence of AF/AT recurrence, and the 2016–2017 group tended to have a lower incidence of AF/AT recurrence than the 2014–2015 group. Although we might not completely have eliminated all the multiple confounders such as the patient characteristics and/or the post‐ablation AADs use, our results not only highlight the importance of the PV durability with balloon ablation or a contact force‐guided ablation,^35^ but also the small effect of extra‐PV‐LA ablation on the maintenance of sinus rhythm in an observational cohort of PerAF patients, as reported in the STAR AF II trial^5^ and another study.^37^

### 4.3 Long‐term clinically relevant outcomes during the recent 6 years

We found a new finding that in both PAF and PerAF, there were only a few incidences of hospitalizations because of HF, major bleeding, and cardiovascular events, strokes/TIAs, and all‐cause mortality without any significant difference among the three time period groups. Numerous studies have demonstrated the potential beneficial effects of sinus rhythm maintenance by catheter ablation on major adverse cardiac and cerebrovascular events.^30^, ^38^, ^39^ Theoretically, PerAF in the 2014–2015 group might have had the worst AF/AT freedom rate, and therefore, the worst clinically relevant events, but no difference was noted among the three time period groups. The clinical impact of ablation may have been dependent on not only AF/AT recurrence, but also the baseline characteristics and post‐ablation AADs and OACs.^40^ The lowest continuation rate of AADs but highest rate of OACs after ablation at the physicians' discretion in the 2016–2017 group may also have reflected the fear of a stroke risk when AF/AT recurrences occur. The high use of post‐ablation OACs (approximately half of the total patients) reflected the current status in Japan.^21^, ^41^ The largest multicenter registries in Japan also showed that over half of the patients had continued OACs at 1 year.^21^, ^41^ In this study, only a few clinically relevant events occurred during the 2‐year follow‐up after AF ablation as reported previously,^20^, ^21^, ^41^ and those incidences were unchanged over the 6 years. Our results suggested that careful post‐ablation management based on the physicians' discretion may be more important for reducing the ultimate clinical outcomes in the remote period after ablation than the recent development of the ablation modalities and strategies in this study cohort during the limited follow‐up duration. Nonetheless, more intensive treatment will be needed in patients at high‐risk for a stroke, because we found a strong association between clinically relevant events and high‐risk patients with a CHADS_2_ score ≥2 or CHA_2_DS_2_‐VASc score ≥3 as previously reported.^21^, ^40^, ^42^

### 4.4 Study limitations

There were several limitations that should be considered. Our study was a retrospective observational single‐center study. Our results may not be generalized to other hospitals, however, our results that did not show any significant effect of extra‐PV‐LA ablation on the maintenance of sinus rhythm in PerAF in the 2014–2015 group, were identical to those in the other RCTs.^5^, ^37^ Second, the frequent use of post‐ablation AADs might have affected our results. For example, the frequent use of post‐ablation bepridil might have led to a higher AF/AT freedom rate than that seen in the previous reports. Nonetheless, the post‐ablation AAD use was strongly associated with a consequence of AF/AT recurrence rather than freedom from AF/AT, and the use of post‐ablation AADs was most frequent in the PerAF patients in the 2014–2015 group, but the incidence of AF/AT recurrence was the highest. Therefore, this effect on the freedom from AF/AF among the three time period groups may be small. Third, over the 6 years, the learning curve of the operators for the technique of the catheter ablation procedure, and/or the operators themselves might have affected our results. Nonetheless, the main operators (Y.O and K.N) were well skilled and were unchanged over the 6 years.

## 5 CONCLUSIONS

This study showed the detailed trends in the ablation modalities and strategies, post‐ablation medication use, and clinical outcomes including clinically relevant events over the recent 6 years. Over the recent 6 years, AF ablation was widely performed in patients who were older and had more PerAF, but the complications decreased. Despite the increase in patients with a remodeled LA and a decrease in the substrate modification strategy, the 2‐year freedom rate from AF/AT recurrence was similar among the three time period groups in the PAF patients. In contrast, despite the lower use of post‐ablation AADs in the PerAF patients, the 2‐year freedom rate from AF/AT recurrence increased in the two more recent time period groups than in the oldest time period group. There were only a few clinically relevant events without a significant difference among the three time period groups. Our findings suggested that the recent development of the ablation modality and strategy provided a favorable impact on sinus rhythm maintenance, especially in PerAF, but their impact on the post‐ablation remote clinical outcomes may have been small in these study patients.

## AUTHOR CONTRIBUTIONS

Moyuru Hirata and Yasuo Okumura wrote the first draft of the protocol article and carries the overall responsibility for the full study and the study protocol. Yasuo Okumura and Koichi Nagashima were substantial contributors to the study concept and design, article drafting, and critical review of the article and will contribute to the acquisition, analysis, and interpretation of the data. Moyuru Hirata, Koichi Nagashima, Ryuta Watanabe, Yuji Wakamatsu, Naoto Otsuka, Satoshi Hayashida, Shu Hirata, Masanaru Sawada, and Sayaka Kurokawa collected the data and conducted the study and have approved the final version of this article. Yasuo Okumura gave us critical comments on the statistical methods and contributed to the analysis and interpretation of the data.

## FUNDING INFORMATION

This work is own‐funded.

## CONFLICT OF INTEREST STATEMENT

The following authors have potential conflicts of interest: YO has received research funding from Bayer Healthcare, Daiichi‐Sankyo, Bristol‐Meyers Squibb, Nippon Boehringer Ingelheim, Pfizer, and Boston Scientific Japan and has accepted remuneration from Bayer Healthcare, Daiichi‐Sankyo, and Bristol‐Meyers Squibb. KN has accepted remuneration from Johnson & Johnson K.K. The other authors have no conflict of interest.

## DECLARATIONS

*Approval of research protocol*: The study was approved by the Institutional Review Board of Nihon University Itabashi Hospital, and an opt‐out system was used to obtain the patients' content for the use of their clinical data for research purposes.

*Date of approval*: February 24, 2023, Approval number: RK‐230214‐9.

*Informed consent*: N/A.

*Registry and the Registration No. of the study/trial*: N/A.

*Animal study*: N/A.

## Supporting information


Figure S1.

Table S1–S2.

## Data Availability

No data are available.
