# Supplementary figures and images for: Trends over the recent 6 years in ablation modalities and strategies, post‐ablation medication, and clinical outcomes of atrial fibrillation ablation
Source: J Arrhythm. 2023 Apr 23;39(3):366–75. doi: 10.1002/joa3.12854 (PMC10264728; doi:10.1002/joa3.12854)

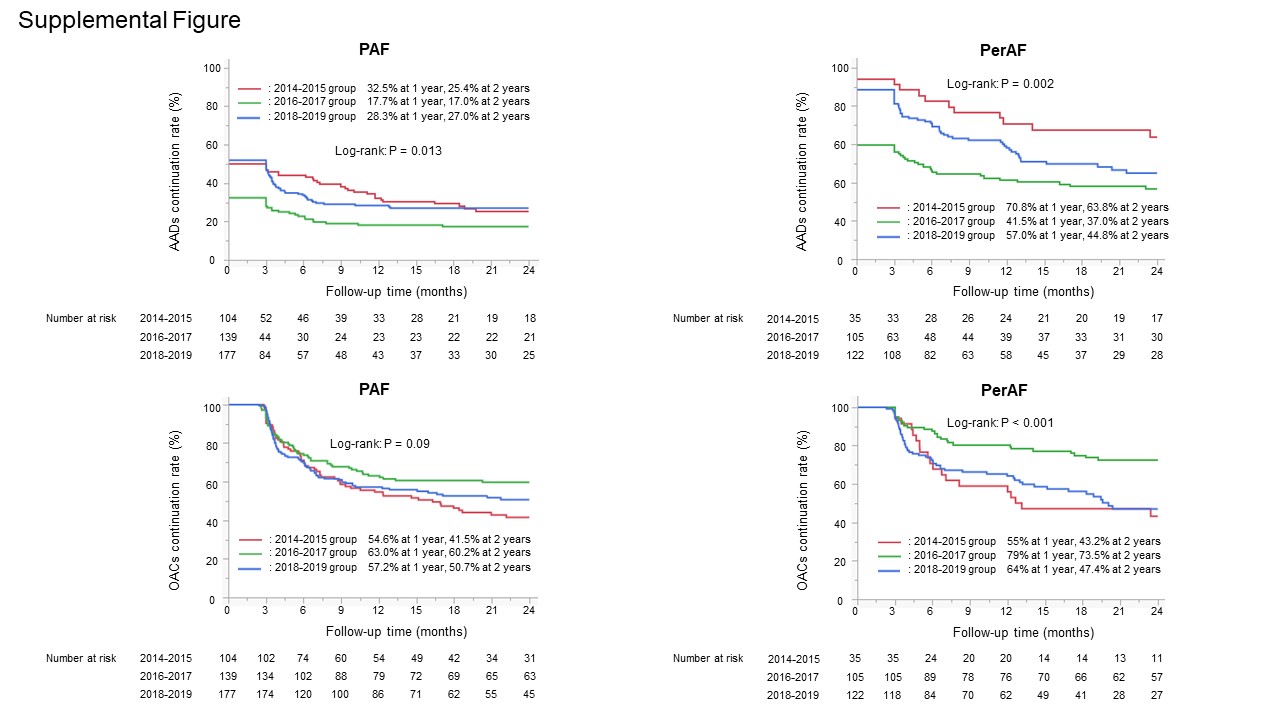

Supplement: Supplementary file 1 — Figure S1. [file JOA3-39-366-s002.jpg]
