# Supplementary material for: Trends over the recent 6 years in ablation modalities and strategies, post‐ablation medication, and clinical outcomes of atrial fibrillation ablation
Source: J Arrhythm. 2023 Apr 23;39(3):366–75. doi: 10.1002/joa3.12854 (PMC10264728; doi:10.1002/joa3.12854)
Supplement: Supplementary file 2 — Table S1–S2. [file JOA3-39-366-s001.docx]

Supplemental Table 1. Patient characteristics, ablation modality, and strategy in the PAF and PerAF patients between the 3 different time periods.

| PAF | | | | |
| --- | --- | --- | --- | --- |
|  | 2014-2015  group  (n=104) | 2016-2017  group  (n=139) | 2018-2019  group  (n=177) | P value |
| Age (years) | 63.5±10.6 | 64.6±9.3 | 67.1±9.8* | 0.006 |
| Male sex | 65 (62.5%) | 97 (69.8%) | 117 (66.1%) | 0.48 |
| BMI (kg/m^2^) | 23.6±4.2 | 24.4±4.1 | 24.0±3.7 | 0.29 |
| Medical history |  |  |  |  |
| HT | 61 (58.7%) | 83 (59.7%) | 98 (55.3%) | 0.71 |
| DM | 14 (13.4%) | 27 (19.4%) | 27 (15.2%) | 0.41 |
| HF | 7 (6.7%) | 14 (10.1%) | 19 (10.7%) | 0.50 |
| Vascular disease | 7 (6.7%) | 7 (5.0%) | 7 (4.0%) | 0.58 |
| Stroke/TIA | 11 (10.6%) | 7 (5.0%) | 17 (9.6%) | 0.21 |
| CHADS_2_ score | 1 (0, 2) | 1 (0, 2) | 1 (0, 2) | 0.71 |
| CHA_2_DS_2_-VASc score | 2 (1, 3) | 2 (1, 3) | 2 (1, 3) | 0.05 |
| Echocardiographic variables |  |  |  |  |
| LVEF (%) | 68.0±8.5 | 68.1±8.8 | 66.6±9.0 | 0.23 |
| LAd (mm) | 37.5±5.6 | 37.9±6.2 | 39.4±6.2* | 0.02 |
| Pre-ablation AADs | 65 (62.5%) | 56 (40.2%)* | 93 (52.5%) | 0.002 |
| Class I | 46 (44.2%) | 41 (29.5%) | 53 (29.9%) | 0.024 |
| Class III | 5 (4.8%) | 4 (2.9%) | 5 (2.8%) | 0.62 |
| Bepridil | 14 (13.5%) | 11 (7.9%) | 37 (20.9%)⁑ | 0.005 |
| β blocker | 19 (18.2%) | 41 (29.5%) | 73 (42.2%)* | <0.001 |
| Modality |  |  |  |  |
| RFCA | 70 (67.3%) | 49 (35.2%)* | 78 (44.1%)* | <0.001 |
| Balloon ablation | 34 (32.69%) | 90 (64.8%)* | 99 (55.9%)* | <0.001 |
| Strategy |  |  |  |  |
| Extra-PV LA ablation | 36 (34.6%) | 13 (9.3%)* | 14 (7.9%)* | <0.001 |
| CTI ablation | 42 (40.3%) | 46 (33.9%) | 46 (25.9%)* | 0.041 |
| PerAF | | | | |
|  | 2014-2015  group  (n=35) | 2016-2017  group  (n=105) | 2018-2019  group  (n=122) | P value |
| Age (years) | 60.0±10.0 | 62.5±11.3 | 63.7±10.7 | 0.19 |
| Male sex | 28 (80%) | 82 (78.1%) | 95 (77.9%) | 0.96 |
| BMI (kg/m^2^) | 25.0±3.3 | 24.8±3.9 | 24.5±3.8 | 0.69 |
| Medical history |  |  |  |  |
| HT | 19 (53.0%) | 63 (60.0%) | 70 (57.4%) | 0.82 |
| DM | 5 (14.3%) | 23 (21.9%) | 13 (10.7%) | 0.06 |
| HF | 3 (8.6%) | 19 (18.1%) | 25 (20.5%) | 0.26 |
| Vascular disease | 1 (2.9%) | 9 (8.6%) | 7 (5.7%) | 0.44 |
| Stroke/TIA | 4 (11.4%) | 11 (10.5%) | 9 (7.4%) | 0.63 |
| CHADS_2_ score | 1 (0, 1) | 1 (0, 2) | 1 (0, 2) | 0.27 |
| CHA_2_DS_2_-VASc score | 2 (1, 2) | 2 (1, 3) | 2 (1, 3) | 0.33 |
| Echocardiographic variables |  |  |  |  |
| LVEF (%) | 66.2±8.1 | 62.1±1.1 | 61.7±10.3 | 0.09 |
| LAd (mm) | 41.5±5.3 | 41.7±6.3 | 43.0±6.8 | 0.23 |
| Pre-ablation AADs | 23 (65.7%) | 43 (40.9%)* | 44 (36.7%)* | 0.007 |
| Class I | 3 (8.6%) | 7 (6.7%) | 8 (6.6%) | 0.91 |
| Class III | 2 (5.7%) | 6 (5.7%) | 4 (3.3%) | 0.64 |
| Bepridil | 18 (51.4%) | 30 (28.6%)* | 35 (28.7%)* | 0.03 |
| β blocker | 8 (22.9%) | 36 (34.3%)* | 56 (45.9%)⁑ | 0.03 |
| Modality |  |  |  |  |
| RFCA | 27 (77.1%) | 38 (36.1%)* | 77 (63.1%)⁑ |  |
| Balloon ablation | 8 (22.9%) | 67 (63.8%)* | 45 (36.9%)⁑ | <0.001 |
| Strategy |  |  |  |  |
| Extra-PV LA ablation | 21 (60.0%) | 9 (8.5%)* | 40 (32.8%) *⁑ | <0.001 |
| CTI ablation | 18 (51.4%) | 38 (36.1%) | 24 (19.6%)⁑ | <0.001 |

PAF, paroxysmal AF; PerAF, persistent AF. Other abbreviations as in Table 1. *P <0.05 vs. 2014-2015 group by the Tukey-HSD test or post-hoc Bonferroni test. ⁑P <0.05 vs. 2016-2017 group by the Tukey-HSD test or post-hoc Bonferroni test.

Supplemental table 2. The details of each adverse event in secondary endpoint

| **All patients** | 2014-2015 group | 2016-2017 group | 2018-2019 group |
| --- | --- | --- | --- |
| Total no.: n=682 | n=139 | n=244 | n=299 |
| Stroke/TIA | 0 | 1 (0.41%) | 1 (0.33%) |
| All-cause mortality | 1 (0.72%) | 4 (2.6%) | 0 |
| Heart failure | 5 (3.6%) | 1 (0.41%) | 0 |
| Major bleeding | 0 | 0 | 2 (0.67%)) |
| Acute coronary syndrome | 2 (1.4%) | 3 (1.2%) | 3 (1.0%) |
| Sick sinus syndrome | 1 (0.72%) | 5 (2.0%) | 2 (0.67%) |
| **Paroxysmal AF** |  |  |  |
| Total no.: n=420 | n=104 | n=139 | n=177 |
| Stroke/TIA | 0 | 1 (0.72%) | 1 (0.56%) |
| All-cause mortality | 0 | 3 (2.1%) | 0 |
| Heart failure | 3 (2.8%) | 0 | 0 |
| Major bleeding | 0 | 0 | 1 (0.56%) |
| Acute coronary syndrome | 2 (1.9%) | 3 (2.1%) | 2 (1.1%) |
| Sick sinus syndrome | 1 (0.96%) | 3 (2.1%) | 2 (1.1) |
| **Persistent AF** |  |  |  |
| Total no.: n=262 | n=35 | n=105 | n=122 |
| Stroke/TIA | 0 | 0 | 0 |
| All-cause mortality | 1 (2.8%) | 1 (0.95%) | 0 |
| Heart failure | 2 (5.7%) | 1 (0.95%) | 0 |
| Major bleeding | 0 | 0 | 1 (0.82%) |
| Acute coronary syndrome | 0 | 0 | 1 (0.82%) |
| Sick sinus syndrome | 0 | 2 (1.9%) | 0 |

The number (%) is shown. AF, atrial fibrillation; TIA, transient ischemic attack.
